# Novel Extraluminal silicone tracheal stent: Development and evaluation in a rabbit model of tracheomalacia

**DOI:** 10.1002/ame2.70062

**Published:** 2025-07-08

**Authors:** Yoon‐Hee Ryu, Chang‐Hwan Moon, Won‐Jong Lee, Jae‐Min Jeong, Hae‐Beom Lee, Seong‐Mok Jeong, Dae‐Hyun Kim

**Affiliations:** ^1^ Department of Veterinary Surgery, College of Veterinary Medicine Chungnam National University Daejeon Republic of Korea; ^2^ Department of Veterinary Surgery, College of Veterinary Medicine Gyeongsang National University Jinju Republic of Korea

**Keywords:** extraluminal tracheal stent, rabbit model, silicone, tracheal collapse, tracheomalacia

## Abstract

Tracheal collapse (TC), defined by excessive tracheal collapsibility, often results in severe respiratory distress in small‐breed dogs. Surgical intervention, including the placement of extraluminal stents, has been employed as a treatment option. Owing to the anatomical and physiological similarities between rabbit and canine tracheas, a rabbit model was utilized to develop a novel extraluminal silicone tracheal stent and evaluate its feasibility in treating tracheomalacia. The stent was surgically implanted in eight New Zealand White rabbits after the induction of tracheomalacia. Postoperative evaluations, including clinical assessment, radiography, computed tomography (CT), and histological analysis, were performed at 1, 2, and 6 months post‐implantation. All rabbits in the stent group survived without exhibiting signs of respiratory distress, whereas all rabbits in the tracheomalacia group experienced respiratory distress, with one succumbing to respiratory failure. Radiographic and CT evaluations confirmed that the stent effectively maintained airway patency, with tracheal measurements not significantly different from the preoperative values, indicating successful restoration of tracheal diameter. Histological analysis demonstrated minimal inflammatory response, the absence of fibrosis, and preserved structural integrity of the tracheal cartilage. Therefore, the novel extraluminal silicone tracheal stent provides effective airway support while minimizing adverse tissue reactions. Further studies, including the use of this stent in a canine TC model and assessment of its long‐term outcomes, are warranted to explore its potential clinical applications in veterinary medicine.

## Graphical abstract

A novel extraluminal silicone stent was developed and applied in a rabbit model of tracheomalacia. The stent effectively maintained airway patency and minimized tissue reaction without causing cartilage damage. This approach may offer a safe and effective alternative for treating tracheal collapse in small animals.

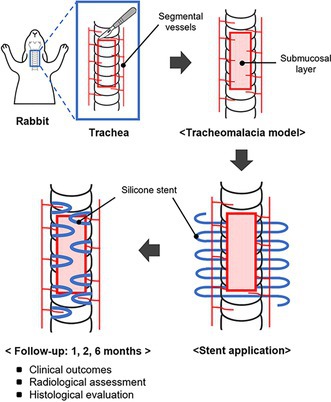

## 1 INTRODUCTION

Tracheal collapse (TC) is a progressive airway disorder commonly seen in small‐ and toy‐breed dogs. It involves dorsoventral flattening of the tracheal lumen, resulting from weakened cartilaginous rings and laxity of the trachealis muscle.^1^, ^2^, ^3^, ^4^ Tracheal collapse in dogs most commonly presents as dorsoventral flattening caused by weakening of the tracheal cartilages and trachealis muscle laxity, although less common morphological variations such as lateral or circumferential collapse have also been described.^5^, ^6^ This anatomical alteration results in dynamic airway obstruction, resulting in chronic coughing, exercise intolerance, and, in severe cases, respiratory distress.^3^, ^7^

Medical management using corticosteroids, bronchodilators, and cough suppressants is commonly employed as the first‐line approach, primarily providing symptomatic relief without addressing the underlying structural degeneration of the trachea.^3^, ^8^, ^9^ When the tracheal lumen is critically narrowed, surgical interventional may be required.^10^

The two primary surgical approaches are intraluminal and extraluminal stenting. Intraluminal stents, placed via fluoroscopic or endoscopy, offer immediate, minimally invasive airway stabilization.^4^, ^11^ However, they carry risks such as infection and granulation tissue formation, leading to secondary airway obstruction.^2^, ^4^, ^12^ Additionally, nitinol stents, commonly used in intraluminal procedures, are prone to fracture.^13^ By contrast, extraluminal stents provide external structural reinforcement of the trachea and reduce the risk of luminal obstruction caused by granulation tissue, although their placement requires a more invasive surgical approach. Despite the availability of these options, long‐term complications such as stent migration, structural failure, and tracheal inflammation continue to present significant challenges.^2^, ^14^ Moreover, the placement of stents may compromise the blood supply, potentially increasing the risk of necrosis, and may damage the recurrent laryngeal nerve, resulting in laryngeal paresis.^3^, ^15^

Various ex vivo and in vivo models have been developed to simulate tracheomalacia and evaluate extraluminal airway splints, including 3D‐printed and bioresorbable devices, which have demonstrated effectiveness in pediatric, porcine, and experimental settings.^16^, ^17^, ^18^, ^19^ Building on these foundations, we developed a segmental tracheomalacia model in rabbits through partial tracheal cartilage resection and evaluated a novel extraluminal silicone stent designed for improved biocompatibility, anatomical conformity, and mechanical support. A rabbit model was chosen for its anatomical similarity to small‐breed dog tracheas and suitability for controlled experiments. The preclinical model enabled refinement of the stent design and assessment of its performance before clinical application in canines.

## 2 MATERIALS AND METHODS

### 2.1 Study design

**FIGURE 1:**
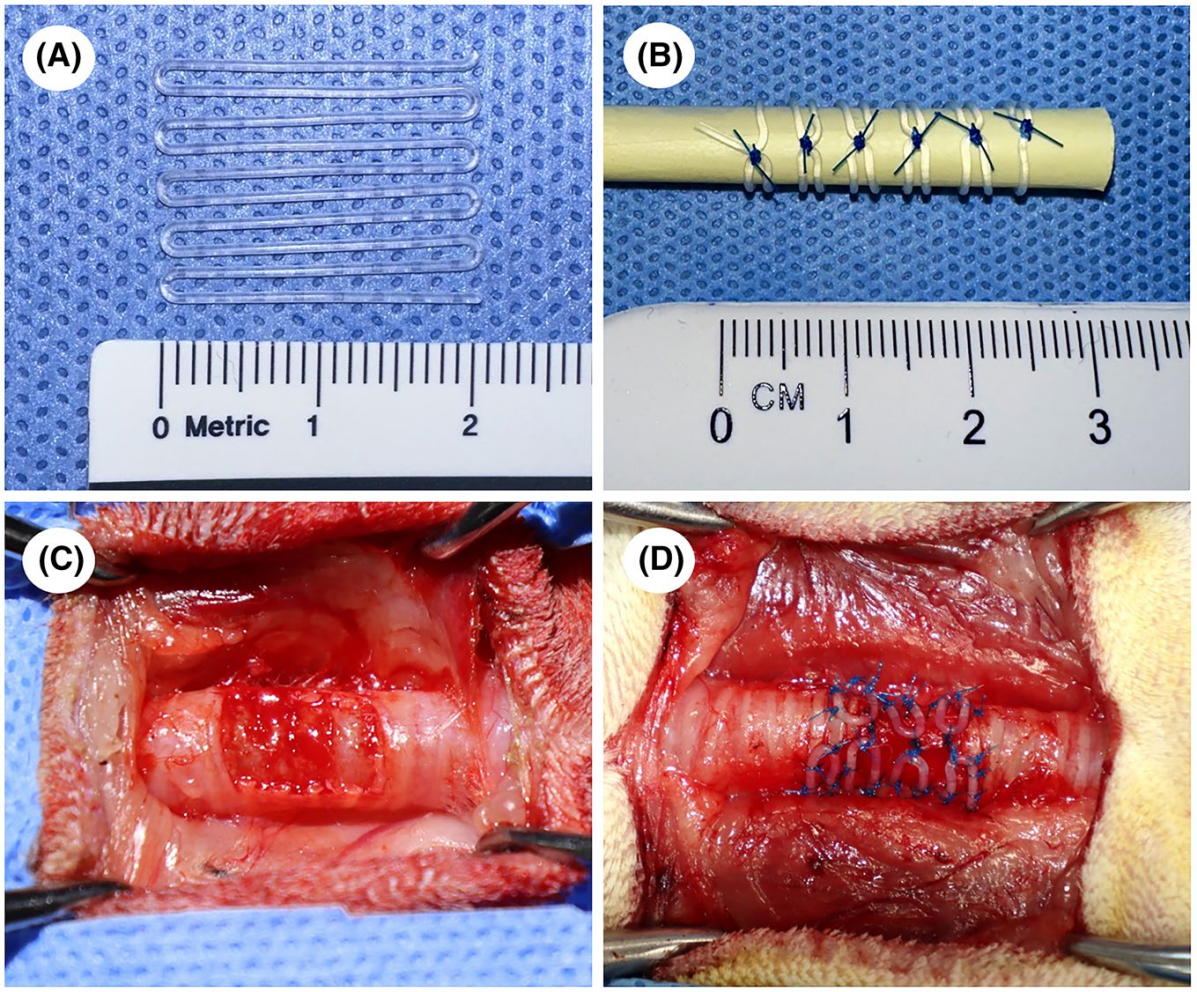
(A) Silicone extraluminal stent fabricated from mold: The stent is made of medical‐grade silicone with a width of 21 mm and a thickness of 0.7 mm. (B) Dorsal view of the rolled‐up silicone stent placed on a tube in which adjacent stent loops were sutured together to maintain cylindrical form, provided for illustrative purposes to aid understanding of the stent's configuration. (C) The tracheomalacia model was established by resecting 50% of the tracheal cartilage over a 1.5‐cm segment, exposing the submucosal layer. (D) The stent was implanted between segmental vessels and the recurrent laryngeal nerve and secured with 6–0 polypropylene sutures.

The stent (Silichem Co., Bucheon, Korea) (Figure 1A,B) was fabricated using a medical‐grade silicone with a hardness of 80. It had a thickness of 0.7 mm and a width of 21 mm, covering the full length of the rabbit trachea.

Eleven specific pathogen‐free (SPF) female New Zealand White rabbits (weighing 3.5–4 kg) were purchased from DooYeol Biotech (Seoul, Korea). Of these, 11 rabbits were assigned to experimental procedures: 3 rabbits underwent partial cartilage resection to induce tracheomalacia (TM group), and 8 rabbits underwent the same procedure followed by extraluminal stent implantation (Stent groups).

Prior to surgical procedures, all 11 rabbits underwent radiographic and CT imaging to obtain baseline tracheal measurements. The preoperative data served as the reference for evaluating changes in tracheal diameter following tracheomalacia induction and stent placement. The stent groups were evaluated at different time points, with three rabbits assigned to the 1‐month (Mo) group, three to the 2‐Mo group, and two to the 6‐Mo group.

All surgical procedures were approved by the Institutional Animal Care and Use Committee of the Clinical Research Institute of Chungnam National University (no. 202312A‐CNU‐198). Animal care and monitoring were carried out by the Chungnam National University Laboratory Animal Research Center.\.

### 2.2 Surgical techniques

Premedication was administered intramuscularly using medetomidine (1 mg/mL; Domitor, Zoetis, Finland) at a dose of 0.6 mg/kg. Anesthesia was induced intravenously using alfaxalone (10 mg/mL; Alfaxan Multidose, Jurox Animal Health, Australia) at a dose of 3 mg/kg. Intubation was performed using a supraglottic airway device (v‐gel®), and anesthesia was maintained with isoflurane (Ifran Liq., Hana Pharm Co., Korea) at a concentration of 3% in oxygen at a flow rate of 1 L/min. Postoperative analgesia was provided through the subcutaneous administration of meloxicam (5 mg/mL; Metacam, Boehringer Ingelheim, Germany) at a dose of 0.3 mg/kg. Rabbits were placed in a dorsal recumbent position with the neck extended. A midline incision was made along the ventral cervical region to expose the underlying structures. The sternohyoid and sternocephalic muscles were separated to access the cervical trachea.

Tracheomalacia was induced by excising approximately 50% of the tracheal cartilage over a 1.5 cm segment of the cervical trachea using a no. 15 blade to expose the submucosal layer, following previously described rabbit models that replicate the segmental collapse observed in canine tracheal collapse (Figure 1C).^20^, ^21^ Collapse of the submucosa in the ventrodorsal direction was confirmed intraoperatively. Following model induction, the overlying muscles were sutured with a 4–0 polydioxanone (Ethicon, Inc.). The subcutaneous tissue was closed, and the skin was sutured with a 4–0 Blue Nylon (Ailee Co.). Postoperative CT imaging was performed under spontaneous respiration, followed by inspiratory phase radiography. Tracheomalacia models were successfully established in three rabbits, and clinical signs—including coughing, goose‐honking sounds, and respiratory distress—were observed and monitored.

The stent model was constructed following the establishment of a tracheomalacia model. The stent was positioned between the segmental vessels and the recurrent laryngeal nerve. Sutures were placed around the annular ligament of the trachea and inserted into the tracheal lumen using a full‐thickness technique. To secure the stent, seven evenly spaced sutures—including the collapsed layer—were placed using 6–0 polypropylene (Prolene, Ethicon, Inc.) ensuring consistent placement in all rabbits (Figure 1D). The surrounding tissues, including the muscle, subcutaneous layers, and skin, were subsequently closed as previously described.

### 2.3 Postoperative clinical evaluation

Clinical signs such as coughing, goose‐honking sounds, and respiratory distress were monitored daily. Respiratory distress was graded based on stridor severity: Grade 0 = no stridor; Grade 1 = intermittent stridor during exertion; Grade 2 = stridor at rest; Grade 3 = continuous biphasic stridor.^22^ Clinical monitoring and respiratory status were recorded for evaluation at 1, 2, and 6 months postoperatively. Postoperative survival was recorded to assess the clinical severity and treatment efficacy.

### 2.4 Radiographic and CT evaluation

Right lateral thoracic radiographs were obtained at full inspiration using a digital radiography system (E7239 X, Toshiba). Cervical tracheal collapse, which was the focus of this model, is most readily observed during inspiration, particularly in the extrathoracic region.^23^ CT scans were acquired using a helical CT scanner (Alexion, Toshiba) under general anesthesia, with rabbits placed in ventral recumbency and the neck extended. Radiographic and CT imaging were performed preoperatively and at 1, 2, and 6 months postoperatively to assess temporal changes in tracheal dimensions and stent performance.

On lateral radiographs, the vertical tracheal diameter (VTD) was measured at the level of the thoracic inlet, corresponding to the caudal aspect of the 7th cervical vertebra.^24^ In CT imaging, the cross‐sectional area (CSA) of the tracheal lumen was measured from transverse slices, with the reference level similarly identified using sagittal views at the caudal aspect of the 7th cervical vertebra. To account for interindividual variation, both VTD and CSA were normalized to their respective values at this anatomical landmark and expressed as ratios.

For preoperative radiographic analysis, the VTD was measured at the caudal body of the 4th cervical vertebra and normalized to the VTD at the caudal aspect of the 7th cervical vertebra. In the TM group, the VTD was measured at the midpoint of the collapsed segment and similarly normalized. In the stented groups, measurements were obtained at the midpoint of the stented segment and normalized in the same manner. This standardized protocol enabled consistent and reliable comparisons of tracheal narrowing across all groups using lateral radiographs.

For preoperative CT evaluation, the CSA was measured from transverse CT images at the caudal portion of the 4th cervical vertebra and normalized to the CSA at the level of the caudal aspect of the 7th cervical vertebra. In the TM group, CSA was measured at the midpoint of the collapsed tracheal segment and normalized accordingly. In the stented groups, CSA was obtained at the midpoint of the stented segment and normalized to the same anatomical reference. This approach ensured consistency and comparability of CT‐based airway measurements across all experimental groups.

Ten veterinarians independently performed all measurements under the supervision of a radiologist, and all observers were blinded to group allocation.

### 2.5 Histopathological evaluation

Histological evaluation was conducted at 1, 2, and 6 months following implantation to compare tissue responses over time. At each designated time point, the rabbits were euthanized under anesthesia through the intravenous injection of potassium chloride (35 mg/kg, JW Pharmaceutical, Korea), and tracheal samples were collected. Tracheal tissues surrounding the stent were collected from the center of the operated segment at each designated time point and fixed in 10% neutral‐buffered formalin. Sections were embedded in paraffin, cut at 4 μm thickness, and stained with Hematoxylin and Eosin (H&E), Masson's trichrome (MT), and Safranin O. Histological scoring of inflammation and fibrosis was performed at 400× magnification in 15 randomly selected fields per rabbit. Two independent veterinary pathologists, blinded to group assignments, conducted the evaluations.

Inflammation was assessed on H&E‐stained slides using two complementary approaches. First, nucleated inflammatory cells were quantitatively counted in 15 randomly selected fields at 400× magnification per rabbit. Second, inflammation severity was graded using a semiquantitative 4‐point scale: 0 = no infiltration, 1 = mild scattered infiltration, 2 = moderate focal dense infiltration, and 3 = severe diffuse infiltration with tissue disruption.^25^ Fibrosis was assessed on MT‐stained sections using a similar 4‐point scale: 0 = no collagen deposition, 1 = mild perivascular or subepithelial fibrosis, 2 = moderate diffuse collagen deposition, and 3 = dense fibrosis with architectural distortion.^25^ Safranin O staining was used to assess cartilage architecture, proteoglycan preservation, mucosal epithelium, and submucosal glandular structures.

### 2.6 Statistical analysis

All statistical analyses were performed using SPSS version 26 (IBM Corp., Armonk, NY, USA). The tracheal diameter was compared between radiographic images (E7239 X, Toshiba) and CT scans (Alexion, Toshiba) to evaluate stent efficacy. Data normality was assessed using the Shapiro–Wilk test. Welch's analysis of variance (ANOVA) was used instead of standard ANOVA. Post‐hoc pairwise comparisons were performed using the Games‐Howell test. The same statistical methods were used for comparisons between radiographic and CT measurements. For histological analysis, H&E staining was used to count nucleated cells and inflammation score, while MT staining was used to assess fibrosis scoring system. One‐way ANOVA was conducted after confirming the normality and homogeneity of variances using Levene's test. A P‐value of less than 0.05 was considered statistically significant, and values less than 0.001 were reported as highly significant.

## 3 RESULTS

### 3.1 Survival and clinical outcomes

All rabbits in the stented groups (1‐Mo, 2‐Mo, and 6‐Mo) survived the observation period without any clinical signs of respiratory distress. Based on the respiratory distress grading scale, all stented animals were classified as Grade 0, indicating normal breathing without abnormal respiratory sounds. In contrast, all rabbits in the tracheomalacia (TM) group developed severe respiratory distress immediately after surgery and were classified as Grade 3, characterized by continuous biphasic stridor. One rabbit in the TM group died within 1 hour postoperatively, resulting in a 66.7% survival rate for this group.

### 3.2 Radiological assessment

**FIGURE 2:**
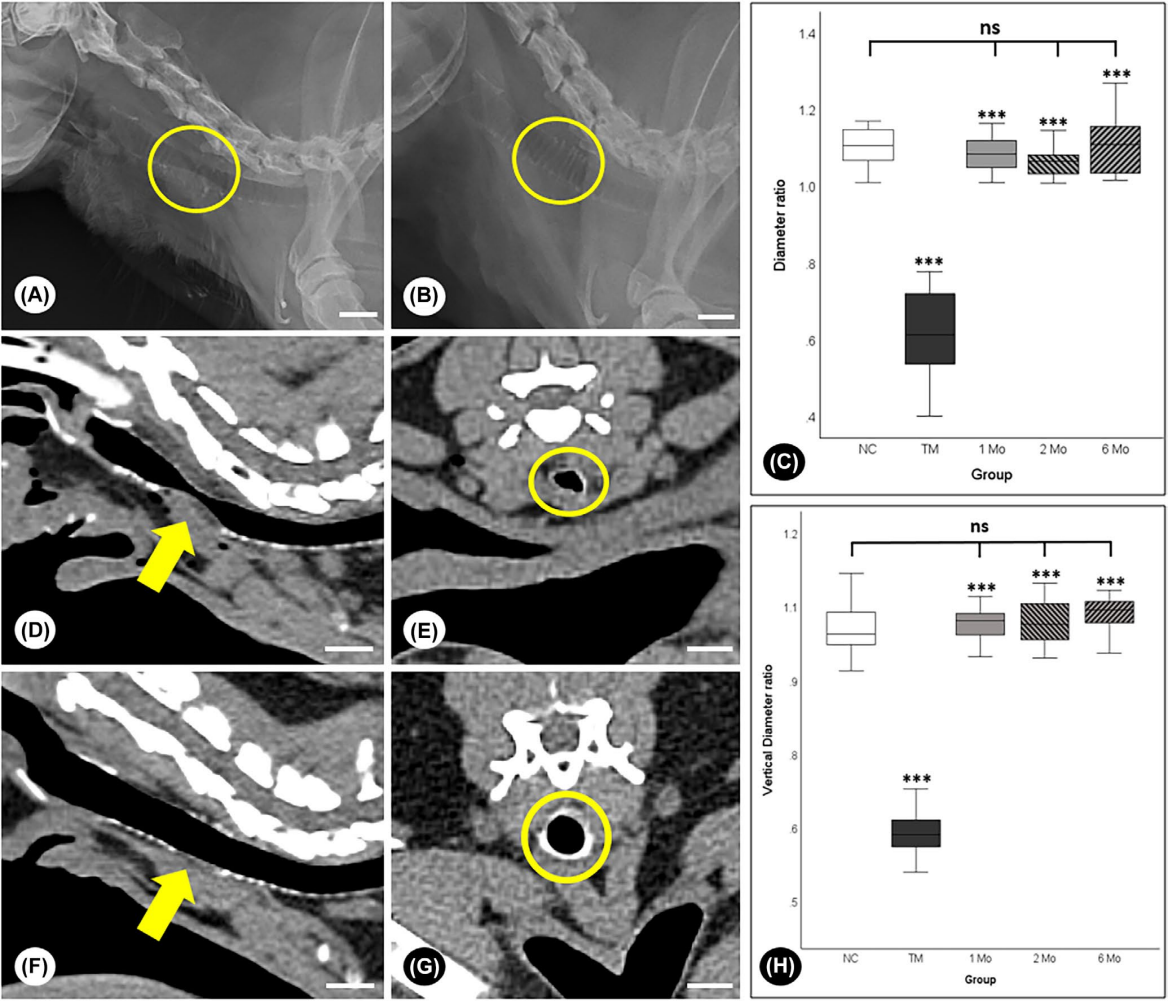
(A) Tracheomalacia model: The yellow circle indicates the collapsed tracheal segment. Magnification: 1x. (B) Stent model (6 months post‐implantation): The yellow circle indicates the implanted stent supporting the trachea. Magnification: 1x. (C) Tracheal diameter ratio: The TM group exhibited the lowest ratio (0.613 ± 0.118), indicating severe collapse, whereas the preoperative value was significantly higher (1.109 ± 0.042). The stented groups demonstrated improved patency (1 Mo: 1.082 ± 0.045, 2 Mo: 1.070 ± 0.044, 6 Mo: 1.110 ± 0.073), and the ratios in the stented groups were comparable to the preoperative value (*p* > 0.05). (D, E) Tracheomalacia model (sagittal and transverse views): The collapsed tracheal segment is visible. (D: Yellow arrow; E: Yellow circle). Magnification: 1x. (F, G) Stent model at 6 months (sagittal and transverse views): The implanted stent provides structural support (F: Yellow arrow; G: Yellow circle). Magnification: 1x. (H) Cross‐sectional area (CSA) ratio: The TM group exhibited the lowest CSA ratio (0.519 ± 0.031), indicating severe airway collapse (*p* < 0.001). In contrast, the stented groups (1 Mo: 1.125 ± 0.011; 2 Mo: 1.165 ± 0.009; 6 Mo: 1.137 ± 0.007) demonstrated significantly improved airway patency, comparable to the preoperative (Pre OP) value (1.133 ± 0.005), suggesting effective structural restoration (*p* > 0.05). ***p < 0.001; ^ns^ *p* > 0.05. Pre OP, preoperative values; TM, tracheomalacia group. Scale bar=10 mm.

Radiograph images were obtained in the right lateral view during full inspiration to evaluate tracheal diameter and the degree of collapse (Figure 2A,B).

In radiographic imaging, the preoperative tracheal diameter ratio was 1.109 ± 0.042. In contrast, the TM group showed the lowest ratio (0.613 ± 0.118), indicating severe airway collapse. The stented groups demonstrated improved tracheal patency, with diameter ratios of 1.082 ± 0.045 at 1 month, 1.070 ± 0.044 at 2 months, and 1.110 ± 0.073 at 6 months (Figure 2C). Statistical analysis confirmed significant differences between the TM group and all other groups (*p* < 0.001). However, the stented groups showed no significant difference from the preoperative ratio (*p* > 0.05), suggesting that stent implantation effectively restored tracheal diameter.

CT imaging provided transverse and sagittal views (Figure 2D–G), along with comprehensive three‐dimensional (3D) assessments of tracheal dimensions. The preoperative cross‐sectional area (CSA) ratio was 1.133 ± 0.005, while the TM group exhibited the lowest value (0.519 ± 0.031), again reflecting severe collapse. The CSA ratios in the stented groups were 1.125 ± 0.011 at 1 month, 1.165 ± 0.009 at 2 months, and 1.137 ± 0.007 at 6 months, demonstrating marked improvement. As with the diameter measurements, significant differences were observed between the TM group and all other groups (*p* < 0.001). The stented groups showed no significant difference from the preoperative CSA ratio (*p* > 0.05), indicating successful airway restoration (Figure 2H).

### 3.3 Histological evaluation

**FIGURE 3:**
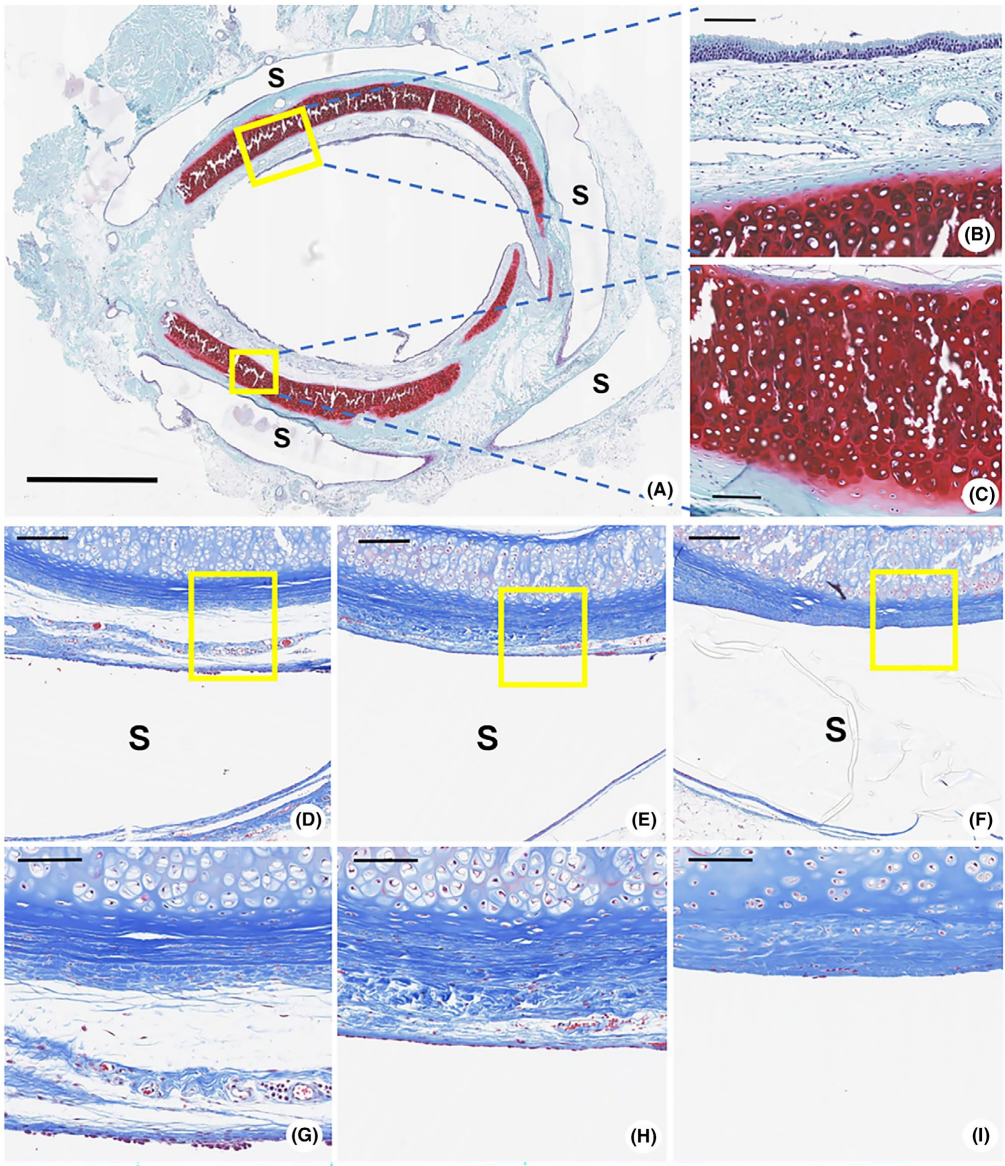
(A–C) Safranin O staining of tracheal tissue from the 6‐month stent group. (A) The stent maintains structural integrity without collapse (black bar = 2000 μm). Magnification: 1x. (B, C) High‐magnification images show intact epithelial cells, glandular structures, and well‐preserved connective tissues, with the cartilage maintaining normal thickness without signs of degeneration (black bar = 100 μm). Magnification: 20x. (D–F) Masson's trichrome staining at 1 month, 2 months, and 6 months (black bar = 200 μm) shows collagen deposition surrounding the stent, with a gradual decrease over time, indicating reduced fibrosis. Magnification: 8x. (G–I) Higher magnification further demonstrates the gradual thinning of collagen (black bar = 100 μm), with the lowest thickness observed at 6 months post‐implantation. Magnification: 20x. S, Stent.

Safranin O staining confirmed intact cartilage structure and proteoglycan preservation, indicating maintenance of tracheal integrity. The mucosal epithelium and glandular structures appeared unaffected, indicating the biocompatibility of the stents (Figure 3A–C). MT staining revealed peak collagen deposition at 1 Mo, a reduction at 2 Mo, and minimal presence at 6 Mo, suggesting a gradual decline in fibrotic response over time (Figure 3D–I). Fibrosis scores declined progressively from 2.3 ± 0.5 (1 Mo) to 1.3 ± 0.6 (2 Mo) and 0.8 ± 0.4 (6 Mo) (*p* < 0.05).

**FIGURE 4:**
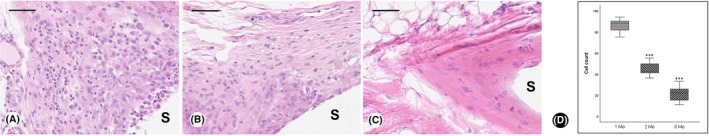
(A–C) Hematoxylin and Eosin staining of the tracheal tissue surrounding the stent at different time points (black bar = 50 μm). (A) A 1‐month stent model: A high degree of inflammatory cell infiltration is observed around the stent. Magnification: 40x. (B) A 2‐month stent model: Inflammatory cell infiltration is reduced compared with that in the 1‐month stent model. Magnification: 40x. (C) A 6‐month stent model: Minimal inflammation indicates adaptation to the stent. Magnification: 40x. (D) Nucleated cell count over time: The nucleated cells were counted in 15 regions. The mean cell count peaked at 1 month (85.80 ± 6.09), declined at 2 months (44.93 ± 5.98), and further decreased at 6 months (21.13 ± 6.66). Statistical analysis confirmed the significant differences across time points (*p* < 0.001). S, stent. ***p < 0.001.

H&E staining was performed to evaluate neovascularization and inflammatory responses at the stent‐tissue interface. Inflammatory cell infiltration was most pronounced at 1 Mo (Figure 4A), decreased at 2 Mo (Figure 4B), and was minimal at 6 Mo (Figure 4C), indicating progressive tissue adaptation. Nucleated cell counts assessed at 15 regions (400×) were highest at 1 Mo (85.80 ± 6.09), were significantly lower at 2 Mo (44.93 ± 5.98), and further reduced 6 Mo (21.13 ± 6.66) (*p* < 0.001) (Figure 4D). In parallel, semiquantitative inflammation scores also decreased significantly over time, from 2.7 ± 0.5 (1 Mo) to 1.7 ± 0.6 (2 Mo) and 0.8 ± 0.4 (6 Mo) (*p* < 0.05), supporting the histological evidence of resolving inflammation.

## 4 DISCUSSION

This study aimed to develop an extraluminal silicone tracheal stent for the treatment of TC in small‐breed dogs and to evaluate its feasibility and biocompatibility in a rabbit tracheomalacia model. The stent effectively maintained airway patency with minimal adverse tissues reactions. Its vascular‐sparing design helps prevent necrosis and nerve damage—common complications of extraluminal supports. Additionally, continuous reinforcement across multiple tracheal rings enhances structural stability.^26^ The silicone's flexibility provides support without excessive pressure, reducing irritation and inflammation, making it a safer alternative to rigid implants.^27^

A rabbit model was selected for its anatomical and physiological similarity to the canine trachea. Its long cervical trachea allows surgical access and mimics TC in small‐breed dogs. Additionally, the segmental blood supply, similar to that in dogs, allows evaluation of vascular responses to extraluminal stent placement.^28^ Previous studies have also demonstrated that the radial force properties of rabbit tracheas are comparable to those of dogs, further supporting their suitability as a preclinical model.^21^ Based on a previously established rabbit tracheomalacia model—achieved by surgically resecting the tracheal rings to induce severe collapse—this model provided a reliable preclinical framework for evaluating extraluminal stents.^20^ Radiographic assessments confirmed the induced tracheomalacia closely and resembled naturally occurring canine TC, reinforcing its translational relevance.

Silicone is widely used in biomedical applications due to its chemical stability, low immunogenicity, and resistance to biodegradation, making it well suited for tracheal stenting.^29^ In intraluminal applications, its mechanical properties provide structural support while maintaining flexibility, allowing conformity to the tracheal wall without exerting excessive pressure or causing irritation.^30^, ^31^ Previous studies have demonstrated that silicone stents exhibit superior biocompatibility compared with metallic stents, reducing the risk of granulation tissue formation and airway trauma.^31^, ^32^ Although most prior research has focused on intraluminal devices, extraluminal silicone stents offer unique advantages by avoiding direct contact with the airway lumen, thereby minimizing mucus retention and stent migration. In this study, the extraluminal silicone stent consistently preserved tracheal patency without signs of mechanical failure or structural deformation. Histological analysis confirmed minimal chronic inflammation and fibrosis over time, with preservation of the underlying cartilage. Notably, at 1 month post‐implantation, inflammation and fibrosis scores were highest, yet no abnormalities were observed on imaging, and clinical respiratory scores remained within normal limits. This discrepancy suggests that early tissue responses may manifest microscopically without corresponding changes in radiographic or functional assessments. These findings underscore the value of histopathological evaluation in revealing subclinical tissue responses and further support the favorable biocompatibility of silicone, which appears to promote localized healing without provoking overt clinical or structural deterioration.

Advancements in 3D printing have expanded the potential of silicone for tracheal stenting. Traditional manufacturing techniques often restrict shape customization; however, 3D printing enables precise control of stent geometry and facilitates the creation of patient‐specific implants.^33^ The stent's simple design is highly compatible with 3D printing, facilitating rapid production and customization. Given the variability in tracheal anatomy, customized stents may improve airway support and minimize complications such as migration and airway obstruction.^34^ Future research should integrate patient‐specific imaging and 3D printing to enhance the precision, adaptability, and clinical efficacy of tracheomalacia treatment.

The study has some limitations, including a small sample size, which may limit the generalizability of the results, and the use of an induced tracheomalacia model that may not fully replicate the pathophysiology and progression of naturally occurring TC. Furthermore, optimizing the stent thickness and length in canine remains crucial for its clinical application. Additionally, extraluminal stent placement requires invasive surgical dissection and may be associated with a longer recovery period compared to intraluminal approaches.

The tracheomalacia model in this study was established by partially resecting the ventral tracheal cartilage while preserving the dorsal membranous wall, thereby inducing dorsoventral collapse—a morphology commonly observed in canine tracheal collapse. Unlike circumferential or posterior collapse, which may arise from more extensive or concentric weakening of the tracheal wall as described in other studies, our method was specifically intended to replicate the crescent‐type deformation frequently encountered in clinical veterinary cases.^17^ While our model was designed to replicate clinically relevant crescent‐type deformities in dogs, we acknowledge that future studies may incorporate modified techniques to induce circumferential or posterior collapse, thereby enabling broader evaluation of stent performance under diverse pathological conditions.

## 5 CONCLUSION

This study demonstrated the feasibility and biocompatibility of a novel extraluminal silicone tracheal stent in a rabbit tracheomalacia model, with the aim of managing TC in small‐breed dogs. The stent effectively maintained airway patency and prevented clinical signs of respiratory distress throughout the study period. Histological evaluation revealed a marked reduction in inflammation and fibrosis over time, with preservation of tracheal cartilage integrity. Its structural design accommodates segmental tracheal vessels, reducing the risk of vascular and neural complications, whereas its flexibility minimizes mechanical irritation. These findings support the structural and biological suitability of silicone for extraluminal stenting. With the potential for patient‐specific customization using 3D printing technology, this stent design may offer a viable therapeutic approach for managing tracheal collapse in dogs. Further validation in canine models is warranted to optimize the design, assess long‐term clinical efficacy, and evaluate its applicability to other collapse morphologies such as circumferential or posterior forms.

## AUTHOR CONTRIBUTIONS

**Yoon‐Hee Ryu:** Conceptualization; formal analysis; investigation; methodology; resources; software; validation; visualization; writing – original draft; writing – review and editing. **Chang‐Hwan Moon:** Data curation; formal analysis; investigation; resources; software; validation; visualization; writing – review and editing. **Won‐Jong Lee:** Formal analysis; investigation; resources; software; validation; visualization; writing – review and editing. **Jae‐Min Jeong:** Data curation; resources; supervision; validation; writing – review and editing. **Hae‐Beom Lee:** Data curation; resources; supervision; validation; writing – review and editing. **Seong‐Mok Jeong:** Data curation; resources; supervision; validation; writing – review and editing. **Dae‐Hyun Kim:** Conceptualization; data curation; methodology; project administration; resources; supervision; validation; visualization; writing – review and editing.

## FUNDING INFORMATION

The authors received no funding for this study.

## CONFLICT OF INTEREST STATEMENT

The authors declare that they have no conflicts of interest.

## ETHICS STATEMENT

All experimental procedures and measurements were approved by the Institutional Animal Care and Use Committee of the Clinical Research Institute, Chungnam National University (no. 202312A‐CNU‐198).

## References

[1] Tangner C , Hobson HP . A retrospective study of 20 surgically managed cases of collapsed trachea. Vet Surg. 1982;11(4):146‐149.

[2] Buback JL , Boothe HW , Hobson HP . Surgical treatment of tracheal collapse in dogs: 90 cases (1983‐1993). J Am Vet Med Assoc. 1996;208(3):380‐384. 8575969

[3] Johnson L . Tracheal collapse: diagnosis and medical and surgical treatment. Vet Clin North Am Small Anim Pract. 2000;30(6):1253‐1266. 11221980 10.1016/s0195-5616(00)06005-8

[4] Durant AM , Sura P , Rohrbach B , Bohling MW . Use of nitinol stents for end‐stage tracheal collapse in dogs. Vet Surg. 2012;41(7):807‐817. doi:10.1111/j.1532-950X.2012.01037.x 22957667

[5] Johnson L , Pollard R . Tracheal collapse and bronchomalacia in dogs: 58 cases (7/2001–1/2008). J Vet Intern Med. 2010;24(2):298‐305. 20051001 10.1111/j.1939-1676.2009.0451.x

[6] Hall E , Baines E , Baines S . Atypical lateral tracheal collapse in a Yorkshire terrier. J Small Anim Pract. 2020;61(10):644‐647. 30387494 10.1111/jsap.12954

[7] Chisnell HK , Pardo AD . Long‐term outcome, complications and disease progression in 23 dogs after placement of tracheal ring prostheses for treatment of extrathoracic tracheal collapse. Vet Surg. 2015;44(1):103‐113. doi:10.1111/j.1532-950X.2014.12206.x 24909184

[8] Tappin SW . Canine tracheal collapse. J Small Anim Pract. 2016;57(1):9‐17. doi:10.1111/jsap.12436 26780854

[9] Payne JD , Mehler SJ , Weisse C . Tracheal collapse. Compend Contin Educ Vet. 2006;28(5):373‐383.

[10] Beranek J , Jaresova H , Rytz U . Use of nitinol self‐expandable stents in 26 dogs with tracheal collapse. Schweiz Arch Tierheilkd. 2014;156(2):91‐98. doi:10.1024/0036-7281/a000555 24463323

[11] Sun F , Usón J , Ezquerra J , Crisóstomo V , Luis L , Maynar M . Endotracheal stenting therapy in dogs with tracheal collapse. Vet J. 2008;175(2):186‐193. 17368061 10.1016/j.tvjl.2007.01.011

[12] Weisse C , Berent A , Violette N , McDougall R , Lamb K . Short‐, intermediate‐, and long‐term results for endoluminal stent placement in dogs with tracheal collapse. J Am Vet Med Assoc. 2019;254(3):380‐392. 30668235 10.2460/javma.254.3.380

[13] Woo H‐M , Kim M‐J , Lee S‐G , et al. Intraluminal tracheal stent fracture in a Yorkshire terrier. Can Vet J. 2007;48(10):1063‐1066. 17987968 PMC1978294

[14] Moser JE , Geels JJ . Migration of extraluminal tracheal ring prostheses after tracheoplasty for treatment of tracheal collapse in a dog. J Am Vet Med Assoc. 2013;243(1):102‐104. 23786197 10.2460/javma.243.1.102

[15] Ayres SA , Holmberg DL . Surgical treatment of tracheal collapse using pliable total ring prostheses: results in one experimental and 4 clinical cases. Can Vet J. 1999;40(11):787‐791. 10563237 PMC1540003

[16] Kaye R , Goldstein T , Aronowitz D , Grande DA , Zeltsman D , Smith LP . Ex vivo tracheomalacia model with 3D‐printed external tracheal splint. Laryngoscope. 2017;127(4):950‐955. 27531619 10.1002/lary.26213

[17] Mondal A , Visner GA , Kaza AK , Dupont PE . A novel ex vivo tracheobronchomalacia model for airway stent testing and in vivo model refinement. J Thorac Cardiovasc Surg. 2023;166(3):679‐687.e1. 37156367 10.1016/j.jtcvs.2023.04.010 PMC10524727

[18] Tsukada H , O'Donnell CR , Garland R , Herth F , DeCamp M , Ernst A . A novel animal model for hyperdynamic airway collapse. Chest. 2010;138(6):1322‐1326. 20651025 10.1378/chest.10-0165

[19] Morrison RJ , Hollister SJ , Niedner MF , et al. Mitigation of tracheobronchomalacia with 3D‐printed personalized medical devices in pediatric patients. Sci Transl Med. 2015;7(285):285ra64‐285ra64. 10.1126/scitranslmed.3010825 PMC4495899 25925683

[20] Gorostidi F , Courbon C , Burki M , Reinhard A , Sandu K . Extraluminal biodegradable splint to treat upper airway anterior malacia: a preclinical proof of principle. Laryngoscope. 2018;128(2):E53‐E58. 28921522 10.1002/lary.26857

[21] Kim JH , Choi JY , Yoon HY . A rabbit model of tracheal collapse for optimal self‐expanding metal stents. J Vet Med Sci. 2023;85(3):386‐392. doi:10.1292/jvms.22-0167 36740259 PMC10076205

[22] Novotny L , Crha M , Rauser P , et al. Novel biodegradable polydioxanone stents in a rabbit airway model. J Thorac Cardiovasc Surg. 2012;143(2):437‐444. 21885070 10.1016/j.jtcvs.2011.08.002

[23] Macready DM , Johnson LR , Pollard RE . Fluoroscopic and radiographic evaluation of tracheal collapse in dogs: 62 cases (2001–2006). J Am Vet Med Assoc. 2007;230(12):1870‐1876. 17571993 10.2460/javma.230.12.1870

[24] Mostafa AA , Berry CR . Radiographic vertical tracheal diameter assessment at different levels along the trachea as an alternative method for the evaluation of the tracheal diameter in non‐brachycephalic small breed dogs. BMC Vet Res. 2022;18(1):61. 35105371 10.1186/s12917-022-03160-4 PMC8805285

[25] Gallet P , Phulpin B , Merlin J‐L , et al. Long‐term alterations of cytokines and growth factors expression in irradiated tissues and relation with histological severity scoring. PLoS One. 2011;6(12):e29399. 22216271 10.1371/journal.pone.0029399 PMC3245280

[26] Suematsu M , Suematsu H , Minamoto T , Machida N , Hirao D , Fujiki M . Long‐term outcomes of 54 dogs with tracheal collapse treated with a continuous extraluminal tracheal prosthesis. Vet Surg. 2019;48(5):825‐834. doi:10.1111/vsu.13229 31115067

[27] Sura PA , Krahwinkel DJ . Self‐expanding nitinol stents for the treatment of tracheal collapse in dogs: 12 cases (2001–2004). J Am Vet Med Assoc. 2008;232(2):228‐236. 18275390 10.2460/javma.232.2.228

[28] Den Hondt M , Vanaudenaerde BM , Delaere P , Vranckx JJ . Twenty years of experience with the rabbit model, a versatile model for tracheal transplantation research. Plast Aesthet Res. 2016;3(7):223. doi:10.20517/2347-9264.2015.117

[29] Habal MB . The biologic basis for the clinical application of the silicones: a correlate to their biocompatibility. Arch Surg. 1984;119(7):843‐848. 6375634 10.1001/archsurg.1984.01390190081019

[30] Théron M‐L , Lahuerta‐Smith T . Laryngeal silicone stent as a treatment option for laryngeal paralysis in dogs: a preliminary study of 6 cases. J Vet Sci. 2022;23(4):e58. 35920122 10.4142/jvs.22068 PMC9346520

[31] Kim D‐H , Choi C‐B , Chung W‐H , et al. Preliminary study for a newly designed silicone stent and delivery system for canine obstructive tracheal disease. J Vet Med Sci. 2012;74(10):1323‐1326. 22673748 10.1292/jvms.11-0448

[32] De Lorenzi D , Maggi G , Bertoncello D , Porciello F , Marchesi MC . Dumon silicone stents can improve respiratory function in dogs with grade IV tracheal collapse: 12 cases (2019–2023). J Am Vet Med Assoc. 2024;1(aop):1‐7. 10.2460/javma.23.12.0722 38569539

[33] Gildea TR , Young BP , Machuzak MS . Application of 3D printing for patient‐specific silicone stents: 1‐year follow‐up on 2 patients. Respiration. 2018;96(5):488‐494. 30212834 10.1159/000489669

[34] Abbas H , Nasim F . Advances in airway stenting. Curr Pulmonol Rep. 2024;13(1):87‐94.

